# Serum zinc levels and in vivo beta-amyloid deposition in the human brain

**DOI:** 10.1186/s13195-021-00931-3

**Published:** 2021-11-19

**Authors:** Jee Wook Kim, Min Soo Byun, Dahyun Yi, Jun Ho Lee, Min Jung Kim, Gijung Jung, Jun-Young Lee, Koung Mi Kang, Chul-Ho Sohn, Yun-Sang Lee, Yu Kyeong Kim, Dong Young Lee, for the KBASE Research Group

**Affiliations:** 1 Department of Neuropsychiatry, Hallym University Dongtan Sacred Heart Hospital, Hwaseong, Gyeonggi 18450 Republic of Korea; 2 Department of Psychiatry, Hallym University College of Medicine, Chuncheon, Gangwan 24252 Republic of Korea; 3 Department of Psychiatry, Pusan National University Yangsan Hospital, Yangsan, 50612 Republic of Korea; 4 Institute of Human Behavioral Medicine, Medical Research Center Seoul National University, Seoul, 03080 Republic of Korea; 5 Department of Geriatric Psychiatry, National Center for Mental Health, Seoul, 04933 Republic of Korea; 6 Department of Neuropsychiatry, Seoul National University Hospital, Seoul, 03080 Republic of Korea; 7 Department of Neuropsychiatry, SMG-SNU Boramae Medical Center, Seoul, 07061 Republic of Korea; 8 Department of Radiology, Seoul National University Hospital, Seoul, 03080 Republic of Korea; 9 Department of Nuclear Medicine, Seoul National University College of Medicine, Seoul, 03080 Republic of Korea; 10 Department of Nuclear Medicine, SMG-SNU Boramae Medical Center, Seoul, 07061 Republic of Korea; 11 Department of Psychiatry, Seoul National University College of Medicine, 101 Daehak-ro, Jongno-gu, Seoul, 03080 Republic of Korea

**Keywords:** Zinc, Aβ, Alzheimer’s disease, APOE4

## Abstract

**Background:**

Despite the known associations between zinc levels and Alzheimer’s disease (AD) dementia and related cognitive impairment, the underlying neuropathological links remain poorly understood. We tested the hypothesis that serum zinc level is associated with cerebral beta-amyloid protein (Aβ) deposition. Additionally, we explored associations between serum zinc levels and other AD pathologies [i.e., tau deposition and AD-signature cerebral glucose metabolism (AD-CM)] and white matter hyperintensities (WMHs), which are measures of cerebrovascular injury.

**Methods:**

A total of 241 cognitively normal older adults between 55 and 90 years of age were enrolled. All the participants underwent comprehensive clinical assessments, serum zinc level measurement, and multimodal brain imaging, including Pittsburgh compound B-positron emission tomography (PET), AV-1451 PET, fluorodeoxyglucose (FDG)-PET, and magnetic resonance imaging. Zinc levels were stratified into three categories: < 80 μg/dL (low), 80 to 90 μg/dL (medium), and > 90 μg/dL (high).

**Results:**

A low serum zinc level was significantly associated with increased Aβ retention. In addition, apolipoprotein E ε4 allele (APOE4) status moderated the association: the relationship between low zinc level and Aβ retention was significant only in APOE4 carriers. Although a low zinc level appeared to reduce AD-CM, the relationship became insignificant on sensitivity analysis including only individuals with no nutritional deficiency. The serum zinc level was associated with neither tau deposition nor the WMH volume*.*

**Conclusions:**

Our findings suggest that decreased serum zinc levels are associated with elevation of brain amyloid deposition. In terms of AD prevention, more attention needs to be paid to the role of zinc.

**Supplementary Information:**

The online version contains supplementary material available at 10.1186/s13195-021-00931-3.

## Background

Zinc is the most abundant trace metal in the brain [1]. Disruption of zinc homeostasis may play a critical role in the pathogenesis of Alzheimer’s disease (AD) [1–3]. Preclinical studies using an AD mouse model revealed that brain zinc bound to beta-amyloid protein (Aβ) plaques and that the levels remained high therein [4] and that zinc treatment increased amyloid precursor protein (APP) expression, enhanced amyloidogenic APP cleavage and Aβ deposition, and impaired spatial learning and memory [5]. A postmortem human brain study revealed that brain zinc accumulation was a prominent feature of AD, linked to brain Aβ accumulation and dementia severity [3]. Serum zinc concentrations from 12 sisters who died in the Nun Study, determined approximately 1 year before death, also showed inverse correlations with senile plaque counts in the brain [6]. Additionally, many human studies have found that serum zinc levels were decreased in AD dementia compared to healthy controls [7–11]. Low serum zinc levels were also associated with rapid progression of AD dementia [2] and poorer cognitive performance [12].

Despite the associations between serum zinc and clinical AD dementia as well as the associations between zinc and Aβ deposition observed in preclinical and postmortem studies, as of yet, no study has investigated the relationship between serum zinc levels and Aβ deposition or other AD-related brain pathologies in the living human brain.

Thus, we aimed to test the hypothesis that serum zinc level is associated with brain Aβ deposition in cognitively normal (CN) older adults. Additionally, we explored the associations between serum zinc level and other AD pathologies (i.e., tau deposition and AD-signature neurodegeneration) and white matter hyperintensities (WMHs), which are measures of cerebrovascular injury.

## Methods

### Participants

This study was part of the Korean Brain Aging Study for Early Diagnosis and Prediction of Alzheimer’s Disease (KBASE), which is an ongoing prospective cohort study [13]. As of February 2017, a total of 241 CN older adults between 55 and 90 years of age were enrolled. The CN group consisted of participants with a Clinical Dementia Rating (CDR) [14] score of 0 and no diagnosis of mild cognitive disorder or dementia. The exclusion criteria were as follows: (1) a major psychiatric illness, (2) a significant neurological (e.g., cerebrovascular) disease or any medical condition that could affect mental function. (3) contra-indications for magnetic resonance imaging (MRI) (e.g., a pacemaker or claustrophobia), (4) illiteracy, (5) the presence of significant visual/hearing difficulties and/or severe communication or behavioral problems that would render clinical examinations or brain scans difficult, and (6) use of an investigational drug. Exclusion criteria were sought by research clinicians who examined laboratory data, MRI results, clinical data collected by trained nurses during systematic interviews of participants, and reliable informants to whom we spoke during screening. More detailed information on recruitment was presented in our previous report [13].

### Clinical assessments

All participants underwent comprehensive clinical and neuropsychological assessments administered by trained psychiatrists and neuropsychologists based on the KBASE assessment protocol [13]. This incorporates the Korean version of the Consortium to Establish a Registry for Alzheimer’s Disease (CERAD) neuropsychological battery [15–17]. Vascular risk factors (e.g., hypertension, diabetes mellitus, dyslipidemia, coronary heart disease, transient ischemic attack, and stroke) were assessed based on data collected by trained nurses during systematic interviews of participants and their family members. A vascular risk score (VRS) was calculated based on the number of vascular risk factors present and reported as a percentage [18]. Body mass index (BMI) was calculated by dividing the weight in kilograms by the square of the height in meters. The Geriatric Depression Scale (GDS) was used to measure the severity of depressive symptoms [19, 20]. Annual income was evaluated and categorized into three groups [below the minimum cost of living (MCL), more than the MCL but below twice the MCL, and twice the MCL or more] (http://www.law.go.kr). The MCL was determined according to the administrative data published by the Ministry of Health and Welfare, Republic of Korea, in November 2012. The MCL was 572,168 Korean Won (KRW) [equivalent to 507.9 US dollars (USD)] per month for single-person households with an additional 286,840 KRW (equivalent to 254.6 USD) per month for each additional housemate. To ensure that the information was accurate, reliable informants were also interviewed.

### Measurement of serum zinc levels and other blood biomarkers

After an overnight fasting, blood samples were obtained via venipuncture in the morning (8–9 a.m.). We measured serum zinc and copper levels using an inductively coupled plasma-mass spectrometer (model 820-MS; Bruker, Australia). We also measured serum copper, calcium, iron, transferrin, and ceruloplasmin levels because they could confound the relationship between zinc levels and brain changes [21]. We measured blood hemoglobin, albumin, and total cholesterol levels to evaluate anemia and nutritional deficiency. Calcium, iron, albumin, and total cholesterol levels were measured using a colorimetric method (ADVIA 1800 Auto analyzer, Siemens, USA). Transferrin and ceruloplasmin were measured employing immunoturbidimetric assays (Cobas Integra 800, Roche Diagnostics). Albumin levels were automatically determined (Advia 1800; Siemens, USA). Hemoglobin levels were determined by flow cytometry (Advia 2120i; Siemens, USA). Genomic DNA was extracted from whole blood and apolipoprotein E (apoE) genotyping performed as previously described [22]. ApoE ε4 allele (APOE4) positivity was defined as the presence of at least one ε4 allele.

### Measurement of cerebral Aβ deposition

All participants underwent contemporaneous three-dimensional (3D) [^11^C] Pittsburgh compound B (PiB)-positron emission tomography (PET) and 3D T1-weighted MRI scanning using a 3.0-T Biograph mMR (PET-MR) platform (Siemens, USA), in accordance with the manufacturer’s guidelines. The PiB-PET imaging acquisition and preprocessing details were described previously [23]. An automatic anatomical labeling algorithm and a region-combining method [24] were applied to determine regions of interest (ROIs) for characterization of PiB retention levels in the frontal, lateral parietal, posterior cingulate-precuneus, and lateral temporal regions. Standardized uptake value ratio (SUVR) values for each ROI were calculated by dividing the mean value for all voxels within each ROI by the mean cerebellar uptake value in the same image. A global cortical ROI consisting of the four ROIs was defined, and a global Aβ retention value was generated by dividing the mean value for all voxels of the global cortical ROI by the mean cerebellar uptake value in the same image [24, 25].

### Measurement of cerebral tau deposition

A subset of subjects (*n* = 58) underwent [^18^F] AV-1451 PET scans using a Biograph True Point 40 PET/CT platform (Siemens, USA), in accordance with the manufacturer’s guidelines. Although all the other neuroimaging scans were performed during the baseline visit, AV-1451 PET imaging was performed at an average of 2.55 (standard deviation = 0.26) years after that visit. The details of AV-1451 PET imaging acquisition and preprocessing have been described previously [23]. To estimate cerebral tau deposition, we quantified the AV-1541 SUVR of an a priori ROI of the “AD-signature region” of tau accumulation, which was a size-weighted average of the partial volume-corrected uptakes by the entorhinal, amygdala, parahippocampal, fusiform, inferior temporal, and middle temporal ROIs, in line with a published method [26]. The AV-1451 SUVR of this ROI served as the outcome variable for cerebral tau deposition.

### Measurement of AD-signature neurodegeneration

All participants underwent [18F] fluorodeoxyglucose (FDG)-PET imaging using the above-described PET-MR platform; the details of FDG-PET image acquisition and preprocessing have been described previously [23]. AD-signature FDG ROIs that are sensitive to the changes associated with AD, such as the angular gyri, posterior cingulate cortex, and inferior temporal gyri [27], were determined. AD-signature cerebral glucose metabolism (AD-CM) was defined as the voxel-weighted mean SUVR extracted from the AD-signature FDG ROIs.

### Measurement of WMH

All participants underwent MRI including T1-weighted images and fluid-attenuated inversion recovery (FLAIR) imaging using the abovementioned 3.0-T PET-MR platform. WMH volume was measured using a validated automatic procedure that has been previously reported [28]. Briefly, the procedure consists of 11 steps: spatial co-registration of T1 and FLAIR images, fusion of T1 and FLAIR images, segmentation of T1 images, acquisition of transformation parameters, deformation and acquisition of the white matter mask, acquisition of FLAIR within the white matter mask, intensity normalization of the masked FLAIR, nomination of candidate WMH with a designated threshold, creation of a junction map, and elimination of the junction. There were two modifications in the current processing procedure relative to that used in the original study: (a) an optimal threshold of 70 was applied, as it was more suitable for our data than the threshold of 65 that was used in the original study; and, (b) given that individuals with acute cerebral infarcts were not enrolled in our sample, we did not use diffusion-weighted imaging in the current automated procedure. Using the final WMH candidate image, WMH volume was extracted in the native space in each subject.

### Statistical analysis

To examine the relationships between the serum zinc level and neuroimaging biomarkers, multiple linear regression analyses were performed as appropriate. The zinc level, which was an independent variable in each analysis, was entered as a stratified categorical variable (three categories: low < 80 μg/dL, medium 80 to 90 μg/dL, and high > 90 μg/dL) by reference to the criteria for zinc deficiency (deficient < 60 μg/dL, marginal deficiency ≥60 to < 80 μg/dL, and normal ≥80 μg/dL) [29]. On statistical analysis, deficiency and marginal deficiency were combined into the low category, because only one participant exhibited a deficiency. Within the normal zinc range, the median value (i.e., 90 μg/dL) served as the cutoff between the low-normal (medium) and high-normal (high) levels. To analyze the associations between serum zinc levels and neuroimaging biomarkers, two models were used for stepwise control of potential confounders. The first model did not include any covariate and the second model included all potential covariates (i.e., age, sex, educational level, APOE4 positivity status, the VRS, the BMI, the GDS score, annual income status, and albumin, copper, calcium, iron, transferrin, and ceruloplasmin levels) that might confound the relationship between the zinc level and brain change [21]. On multiple linear regression analyses, global Aβ retention and WMH values were subjected to natural log-transformations to achieve normal distributions. In all analyses, a high zinc level served as the reference (i.e., high zinc vs. medium zinc, or high zinc vs. low zinc). Sensitivity analyses were performed for the participants with no nutritional deficiencies (i.e., serum albumin < 3.5 g/dL and total cholesterol < 150 mg/dL) [30, 31] to reduce the influence of a general nutritional deficiency on the association between serum zinc level and brain changes. To explore the influence of age, sex, APOE4 positivity, VRS, and the copper, calcium, and iron levels on the associations between serum zinc levels and the biomarkers that were significant in the analyses described above, the regression analyses were repeated but now including two-way interaction terms between serum zinc levels and the biomarkers as additional independent variables. All statistical analyses were performed with the aid of IBM SPSS Statistics software (version 27, IBM Corp., Armonk, NY, USA).

## Results

### Participant characteristics

The demographic and clinical characteristics of all participants are presented in Table 1. Of the total of 241 participants, 70 had low zinc levels, 80 had medium zinc levels, and 91 had high zinc levels.

**Table 1:** Demographic and clinical characteristics of cognitively normal participants by the categories of serum zinc

Characteristic	Overall	Categorized zinc level			*P*
		Low	Medium	High	
*N*	241	70	80	91	
Age, y	69.73 (8.00)	70.14 (7.13)	71.04 (8.41)	68.26 (8.11)	0.067^a^
Female, *n* (%)	114 (47.30)	36 (51.43)	39 (48.75)	39 (42.86)	0.531^b^
Education, y	11.61 (4.76)	11.00 (4.84)	11.39 (4.92)	12.27 (4.50)	0.212^a^
APOE4 positivity, *n* (%)	44 (18.26)	14 (20.00)	15 (18.75)	15 (16.48)	0.841^b^
VRS, %	17.57 (16.47)	16.90 (14.33)	18.54 (17.18)	17.22 (17.47)	0.806^a^
MMSE	26.82 (2.67)	26.34 (2.44)	26. 93 (3.29)	27.10 (2.18)	0.189^a^
BMI, kg/m^2^	24.18 (2.92)	24.29 (3.06)	24.00 (2.80)	24.25 (2.94)	0.793^a^
GDS score	4.93 (5.09)	5.04 (5.24)	5.63 (5.26)	4.22 (4.76)	0.192^a^
Annual income status					0.887^c^
< MCL, *n* (%)	15 (6.22)	3 (4.29)	5 (6.25)	7 (7.69)	
≥MCL, < 2 × MCL, *n* (%)	115 (47.72)	32 (45.71)	40 (50.00)	43 (47.25)	
≥2 × MCL, *n* (%)	111 (46.06)	35 (50.00)	35 (43.75)	41 (45.05)	
Zinc, μg/dL	86.85 (11.79)	73.36 (4.90)	85.04 (2.85)	98.80 (7.50)	< 0.001^a^
Albumin, g/dL	4.49 (0.25)	4.38 (0.23)	4.53 (0.21)	4.53 (0.27)	< 0.001^a^
Copper, μg/dL	102.32 (17.78)	100.73 (20.25)	101.91 (15.62)	103.91 (17.60)	0.515^a^
Calcium, mg/dL	9.45 (0.31)	9.35 (0.30)	9.46 (0.29)	9.50 (0.32)	0.010^a^
Iron, μg/dL	122.61 (40.75)	111.59 (41.13)	116.84 (38.35)	136.18 (39.13)	< 0.001^a^
Transferrin, mg/dL	271.15 (39.38)	262.74 (37.04)	271.26 (38.67)	277.51 (40.92)	0.061^a^
Ceruloplasmin, mg/dL	24.14 (5.08)	23.91 (5.78)	24.87 (4.41)	23.67 (5.04)	0.276^a^
Total cholesterol, mg/dL	183.57 (31.49)	181.50 (27.66)	180.75 (28.86)	187.64 (36.05)	0.293^a^
Nutritional deficiency	36 (14.94)	10 (14.29)	13 (16.25)	13 (14.29)	0.922^b^
Neuroimage biomarkers					
Cerebral Aβ deposition					
Aβ retention, SUVR	1.20 (0.23)	1.26 (0.34)	1.20 (0.21)	1.16 (0.12)	0.021^a^
Cerebral tau deposition (*n* = 58)					
AV-1451, SUVR	1.34 (0.26)	1.36 (0.30)	1.29 (0.21)	1.38 (0.24)	0.575^a^
AD-CM, SUVR	1.42 (0.12)	1.39 (0.13)	1.41 (0.12)	1.44 (0.12)	0.077^a^
WMH volume, cm^3^	12.29 (11.68)	11.14 (10.13)	12.91 (12.37)	12.65 (12.25)	0.625^a^

Abbreviations: *MMSE* Mini-Mental State Examination, *APOE4* apolipoprotein E ε4 allele, *VRS* vascular risk score, *BMI* body mass index, *GDS* geriatric depression scale, *MCL* minimum cost of living, *Aβ* beta-amyloid, *AD* Alzheimer’s disease, *AD-CM* Alzheimer’s disease signature cerebral glucose metabolism, *SUVR* standardized uptake value ratio

Data are expressed as mean (standard deviation), unless otherwise indicated

^a^By one-way analysis of variance

^b^By chi-square test

^c^By Fisher exact test

### Association of serum zinc level with cerebral Aβ deposition

**Fig. 1:**
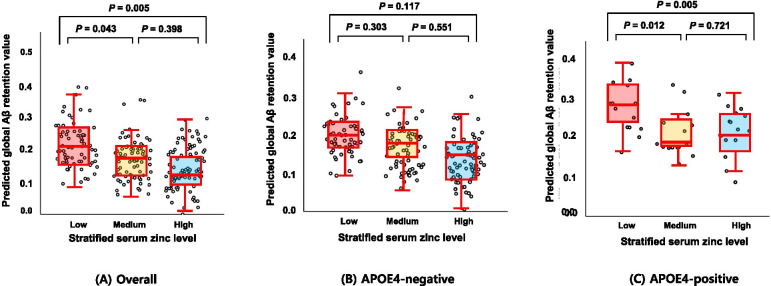
Box plots displaying stratified serum zinc levels and global Aβ retentions in cognitively normal older participants. **A** Overall and **B**, **C** by subgroup [**B** APOE4-negative and **C** APOE4-positive]. Footnotes: Error bars indicate standard errors. Multiple linear regression analyses were performed after adjusting for all confounding factors

Low serum zinc was associated with significantly increased global Aβ retention compared to the high serum zinc (Table 2 and Fig. 1). Additional analyses for the association between serum zinc and regional Aβ retention showed similar results (Table 3), indicating no regional specificity of the association. Sensitivity analysis of participants with no nutritional deficiencies yielded similar results (Table 4).

**Table 2:** The results of multiple linear regression analyses assessing the relationships between the serum zinc strata and the Aβ, AV-1451, AD-CM, and WMH status of cognitively normal older adults

	Stratified zinc level				
	Low		Medium		High
	*B* (*95% CI*)	*P*	*B* (*95% CI*)	*P*	
Aβ retention, SUVR					
Model 1	0.064 (0.015 to 0.113)	0.011	0.025 (−0.022 to 0.073)	0.291	Reference
Model 2	0.072 (0.022 to 0.123)	0.005	0.020 (−0.027 to 0.068)	0.398	Reference
AV-1451, SUVR					
Model 1	−0.020 (− 0.195 to 0.154)	0.817	−0.090 (− 0.277 to 0.097)	0.340	Reference
Model 2	0.023 (−0.179 to 0.225)	0.819	−0.019 (− 0.233 to 0.195)	0.859	Reference
AD-CM, SUVR					
Model 1	−0.045 (− 0.083 to − 0.006)	0.025	− 0.024 (− 0.061 to 0.014)	0.213	Reference
Model 2	−0.048 (− 0.091 to − 0.006)	0.024	− 0.020 (− 0.059 to 0.019)	0.317	Reference
WMH, cm^3^					
Model 1	−0.070 (− 0.381 to 0.241)	0.656	−0.012 (− 0.317 to 0.293)	0.939	Reference
Model 2	−0.177 (− 0.504 to 0.149)	0.285	−0.089 (− 0.394 to 0.216)	0.567	Reference

Abbreviations: *Aβ* beta-amyloid, *AD-CM* Alzheimer’s disease signature cerebral glucose metabolism, *CI* confidence interval, *APOE4* apolipoprotein E ε4 allele, *VRS* vascular risk score, *BMI* body mass index, *GDS* geriatric depression scale

The results of multiple linear regression analyses are presented with *B* coefficient values, *95% CI*, and *P* value. Global Aβ retention and WMH values were used after natural log-transformation to achieve normal distribution. Model 1 did not include any covariates and model 2 included all potential covariates, including age, sex, education, APOE4 positivity, VRS, BMI, GDS score, annual income status, albumin, copper, calcium, iron, transferrin, and ceruloplasmin

**Table 3:** Results of multiple linear regression analyses for assessing the relationship between stratified zinc level and sub-regional Aβ deposition in cognitively normal individuals

	Frontal region		PC-PRC region		Lt. parietal region		Lt. temporal region	
	*B* (*95% CI*)	*P*	*B* (*95% CI*)	*P*	*B* (*95% CI*)	*P*	*B* (*95% CI*)	*P*
Model 1^a^								
Low	0.100 (0.028 to 0.173)	0.007	0.128 (0.034 to 0.221)	0.008	0.1145 (0.035 to 0.192)	0.005	0.095 (0.028 to 0.161)	0.005
Medium	0.035 (− 0.035 to 0.105)	0.326	0.050 (− 0.040 to 0.141)	0.275	0.043 (− 0.032 to 0.119)	0.260	0.038 (−0.026 to 0.102)	0.243
High	Reference		Reference		Reference		Reference	
Model 2^b^								
Low	0.116 (0.041 to 0.192)	0.003	0.148 (0.050 to 0.246)	0.003	0.131 (0.049 to 0.212)	0.002	0.112 (0.043 to 0.181)	0.002
Medium	0.031 (−0.040 to 0.101)	0.392	0.044 (− 0.048 to 0.135)	0.350	0.041 (−0.036 to 0.117)	0.297	0.037 (−0.027 to 0.101)	0.260
High	Reference		Reference		Reference		Reference	

Abbreviations: *Aβ* beta-amyloid, *CI* confidence interval, *APOE4* apolipoprotein E ε4 allele, *VRS* vascular risk score, *BMI* body mass index, *GDS* geriatric depression scale

The results of multiple linear regression analyses are presented with *B* coefficient values, *95% CI*, and *P* value. Global Aβ retention was used after natural log-transformation to achieve normal distribution. Model 1 did not include any covariates and model 2 included all potential covariates, including age, sex, education, APOE4 positivity, VRS, BMI, GDS score, annual income status, albumin, copper, calcium, iron, transferrin, and ceruloplasmin

**Table 4:** The results of multiple linear regression analyses assessing the relationships between the serum zinc strata and the Aβ, AV-1451, AD-CM, and WMH status of cognitively normal older adults with no nutritional deficiencies

	Stratified zinc level				
	Low		Medium		High
	*B* (*95% CI*)	*P*	*B* (*95% CI*)	*P*	
Aβ retention, SUVR					
Model 1	0.065 (0.011 to 0.120)	0.020	0.027 (− 0.026 to 0.081)	0.310	Reference
Model 2	0.082 (0.025 to 0.138)	0.005	0.034 (−0.020 to 0.089)	0.217	Reference
AV-1451, SUVR					
Model 1	−0.021 (− 0.213 to 0.171)	0.827	−0.085 (00.296 to 0.127)	0.425	Reference
Model 2	0.010 (−0.221 to 0.240)	0.933	−0.050 (− 0.313 to 0.212)	0.699	Reference
AD-CM, SUVR					
Model 1	−0.032 (− 0.074 to 0.010)	0.134	−0.016 (− 0.057 to 0.025)	0.436	Reference
Model 2	−0.033 (− 0.078 to 0.013)	0.162	−0.010 (− 0.054 to 0.034)	0.668	Reference
WMH, cm^3^					
Model 1	− 0.078 (− 0.426 to 0.271)	0.660	−0.067 (− 0.408 to 0.275)	0.701	Reference
Model 2	−0.215 (− 0.585 to 0.156)	0.254	−0.148 (− 0.503 to 0.207)	0.411	Reference

Abbreviations: *Aβ* beta-amyloid, *AD-CM* Alzheimer’s disease signature cerebral glucose metabolism, *CI* confidence interval, *APOE4* apolipoprotein E ε4 allele, *VRS* vascular risk score, *BMI* body mass index, *GDS* geriatric depression scale

The results of multiple linear regression analyses are presented with *B* coefficient values, *95% CI*, and *P* value. Global Aβ retention and WMH values were used after natural log-transformation to achieve normal distribution. Model 1 did not include any covariates and model 2 included all potential covariates, including age, sex, education, APOE4 positivity, VRS, BMI, GDS score, annual income status, albumin, copper, calcium, iron, transferrin, and ceruloplasmin

### Association of the zinc level with other brain pathologies

No differences in terms of tau deposition or WMH were observed between the serum zinc categories (Tables 2 and 4). A reduced AD-CM was related to a low serum zinc level (Table 2), but the relationship became insignificant on sensitivity analysis of only individuals with no nutritional deficiency (Table 4).

### Moderation of the association between the serum zinc level and cerebral Aβ deposition

The serum zinc × APOE4 positivity interaction was significant in terms of Aβ retention, indicating that APOE4 positivity moderates the association between the serum zinc level and cerebral Aβ deposition (Table 5). Further subgroup analyses showed that low serum zinc was significantly associated with higher Aβ deposition in the APOE4-positive but not APOE4-negative subgroup (Table 6 and Fig. 1). The interactions between the serum zinc level and other variables, including sex, the VRS, and copper, calcium, and iron levels, were not significant (Table 5).

**Table 5:** The results of multiple linear regression analyses including interaction terms between the serum zinc strata and age (or sex, or APOE4 positivity, or the VRS, or the copper level) in terms of predicting Aβ retention

	*B* (*95% CI*)^a^	*P*
Low zinc	0.056 (− 0.176 to 0.288)	0.636
Medium zinc	0.119 (−0.061 to 0.299)	0.193
Age	0.006 (0.003 to 0.009)	< 0.001
Low zinc × age	< 0.001 (−0.003 to 0.004)	0.913
Medium zinc × age	−0.001 (− 0.004 to 0.001)	0.250
Low zinc	0.047 (−0.020 to 0.114)	0.170
Medium zinc	0.029 (−0.035 to 0.093)	0.374
Sex	0.002 (− 0.063 to 0.067)	0.953
Low zinc × sex	0.055 (− 0.038 to 0.149)	0.247
Medium zinc × sex	−0.021 (− 0.111 to 0.068)	0.637
Low zinc	0.040 (−0.015 to 0.096)	0.152
Medium zinc	0.016 (− 0.036 to 0.067)	0.553
APOE4 positivity	−0.002 (− 0.086 to 0.082)	0.958
Low zinc × APOE4 positivity	0.162 (0.040 to 0.284)	0.010
Medium zinc × APOE4 positivity	0.030 (−0.089 to 0.148)	0.624
Low zinc	0.101 (0.029 to 0.174)	0.006
Medium zinc	0.051 (−0.015 to 0.117)	0.130
VRS	0.008 (−0.023 to 0.040)	0.607
Low zinc × VRS	−0.029 (− 0.080 to 0.022)	0.260
Medium zinc × VRS	−0.028 (− 0.071 to 0.015)	0.197
Low zinc	0.060 (−0.128 to 0.249)	0.530
Medium zinc	0.049 (−0.111 to 0.209)	0.547
Copper	< 0.001 (−0.002 to 0.002)	0.912
Low zinc × copper	< 0.001 (−0.002 to 0.002)	0.904
Medium zinc × copper	< 0.001 (−0.002 to 0.001)	0.699
Low zinc	0.049 (−0.222 to 0.320)	0.719
Medium zinc	0.112 (−0.101 to 0.324)	0.300
Calcium	0.050 (−0.022 to 0.122)	0.175
Low zinc × calcium	0.002 (−0.027 to 0.031)	0.877
Medium zinc × calcium	−0.010 (− 0.033 to 0.012)	0.365
Low zinc	0.019 (−0.120 to 0.158)	0.788
Medium zinc	0.059 (−0.066 to 0.184)	0.351
Iron	< 0.001 (< 0.001 to 0.001)	0.445
Low zinc × iron	< 0.001 (−0.001 to 0.002)	0.390
Medium zinc × iron	< 0.001 (−0.001 to 0.001)	0.451

Abbreviations: *APOE4* apolipoprotein ε4, *VRS* vascular risk score, *Aβ* beta-amyloid, *CI* confidence interval, *APOE4* apolipoprotein E ε4 allele, *VRS* vascular risk score

^a^Multiple linear regression model included zinc, age (or sex or APOE4 positivity or VRS or copper or calcium or iron), and the interaction between zinc and age (or sex or APOE4 positivity or VRS or copper or calcium or iron) treated as the independent variables; for all potential confound factors were treated as covariates; and Aβ treated as the dependent variable

**Table 6:** The results of the multiple linear regression analyses of the relationships between the serum zinc strata and Aβ retention in terms of APOE4 positivity

	Stratified zinc level				
	Low		Medium		High
	*B* (*95% CI*)	*P*	*B* (*95% CI*)	*P*	
APOE4-positive					
Model 1	0.184 (0.031 to 0.337)	0.020	0.021 (−0.130 to 0.171)	0.783	Reference
Model 2	0.242 (0.079 to 0.406)	0.005	0.027 (−0.128 to 0.182)	0.721	Reference
APOE4-negative					
Model 1	0.032 (−0.016 to 0.080)	0.193	0.026 (−0.021 to 0.072)	0.274	Reference
Model 2	0.040 (−0.010 to 0.091)	0.117	0.014 (−0.033 to 0.061)	0.551	Reference

Abbreviations: *Aβ* beta-amyloid, *APOE4* apolipoprotein ε4, *CI* confidence interval, *APOE4* apolipoprotein E ε4 allele, *VRS* vascular risk score, *BMI* body mass index, *GDS* geriatric depression scale

The results of multiple linear regression analyses are presented with *B* coefficient values, *95% CI*, and *P* value. Global Aβ retention values were used after natural log-transformation to achieve normal distribution. Model 1 did not include any covariates and model 2 included all potential covariates, including age, sex, education, APOE4 positivity, VRS, clinical diagnosis, BMI, GDS score, annual income status, albumin, copper, calcium, iron, transferrin, and ceruloplasmin

## Discussion

In older individuals lacking cognitive impairment, low serum zinc was associated with increased in vivo global Aβ retention, supporting the hypothesis that low serum zinc is associated with high brain amyloid deposition, in line with the inverse correlation between serum zinc levels determined about 1 year before death and the postmortem senile plaque counts in 12 individuals of the Nun Study [6]. Many clinical studies have also reported associations between low serum zinc levels and clinical AD dementia or poor cognitive performance.

Regarding the mechanism underlying the relationship between lower serum zinc and higher brain Aβ deposition, lower serum zinc may reflect sequestration of zinc in the brain due to its binding to Aβ and depletion in other body compartments such as blood [6, 32]. Zinc was bound to Aβ plaques and remained high in such plaques in an AD mouse model [4], and senile plaques were markedly enriched with zinc in the human brain [33, 34]. A preclinical study also demonstrated that human Aβ peptide specifically bound zinc [35]. Although the consequence of zinc sequestration by Aβ peptides and its impact on AD pathogenesis is not clearly understood yet, recent evidences suggest that decreased zinc levels in the synaptic cleft can alter glutamatergic excitotoxic neurotransmission and promote synaptic failure and neuronal death [32, 35–37]. Alternatively, lower serum zinc level caused by dietary zinc deficiency might result in more Aβ deposition, as has been demonstrated in an animal model study [38]. However, this possibility seems not so high given that the sensitivity analysis for individuals with no nutritional deficiency revealed similar results.

We also found that APOE4 status moderated the relationship between the serum zinc level and amyloid deposition. We found a significant negative association between the zinc level and Aβ deposition in participants with APOE4, but not those without APOE4. This may reflect associative interactions between zinc, the apoE4 isoform, and Aβ deposition [39]. ApoE4 binds to Aβ and facilitates Aβ fibrillation more easily than do the apoE2 and apoE3 isoforms [40, 41]. Additionally, as the sulfhydryl groups of cysteine residues are responsible for zinc binding [42, 43], the arginine substitutions in apoE4 restrict its ability to control zinc homeostasis and zinc-dependent molecular changes in the AD brain [44].

We additionally found a significant association between a low serum zinc level and decreased AD-CM. In contrast, we found no association between the serum zinc level and either tau deposition or WMH, indicating that zinc-related hypometabolism is possibly not mediated by brain tau deposition or cerebrovascular injury. The interaction of zinc with Aβ within Aβ plaques in the AD brain may trigger a neuronal zinc imbalance at the glutamatergic synapse level, further exacerbating synaptic dysfunction and the associated cerebral hypometabolism [1, 11, 45, 46]. Another possible explanation for the association between a low serum zinc level and brain hypometabolism is that the association may be cofounded by nutritional status; a nutritional deficiency is associated with both low serum zinc levels and reduced brain metabolism [47]. Sensitivity analysis of only participants lacking nutritional deficiencies revealed that the association between the serum zinc level and AD-CM was no longer significant, supporting the above explanation.

## Limitations

To the best of our knowledge, this study is the first to show an association between the serum zinc level and brain Aβ accumulation in living humans. Even after controlling for all potential confounders, the findings did not change. The results were confirmed by sensitivity analysis performed after excluding participants with nutritional deficiencies. However, our study had a couple of limitations. First, as this was a cross-sectional work, causal relationships cannot be inferred. Long-term prospective studies are needed. Second, tau PET was performed at an average of 2.55 years (standard deviation 0.26 years) after the baseline visit; the other neuroimaging scans were performed at baseline. This temporal gap may have influenced the association between zinc and tau. When we controlled for the temporal gap as an additional covariate, however, the results did not change. In addition, only a subset of participants (*n* = 58) underwent tau PET, whereas all participants underwent all other imaging modalities. The lower tau PET sample size may have decreased the statistical power and thus contributed to the null result for the relationship between zinc level and tau deposition. A study with a larger sample size is required.

## Conclusion

The present findings suggest that a decreased serum zinc level is associated with elevation of brain amyloid deposition, particularly in APOE4 carriers. In terms of AD prevention, more attention needs to be paid to the role of zinc.

## Supplementary Information

**Additional file 1**: Supplementary material including authors list for the KBASE group.

## Data Availability

The data of the current study are not freely accessible because the Institutional Review Board (IRB) of Seoul National University Hospital prohibits public data-sharing for privacy reasons. However, the data may be available from the independent data-sharing committee of the KBASE research group on reasonable request, after approval by the IRB. Requests for data access can be submitted to the administrative coordinator of the KBASE group by e-mail (kbasecohort@gmail.com); the coordinator is independent of the authors.
