# Supplementary material for: Serum zinc levels and in vivo beta-amyloid deposition in the human brain
Source: Alzheimers Res Ther. 2021 Nov 19;13:190. doi: 10.1186/s13195-021-00931-3 (PMC8605596; doi:10.1186/s13195-021-00931-3)
Supplement: Supplementary file 1 — Additional file 1: Supplementary material including authors list for the KBASE group. [file 13195_2021_931_MOESM1_ESM.docx]

**Authors**

| Name | Location | Role | Contribution |
| --- | --- | --- | --- |
| Jee Wook Kim, MD, PhD | Hallym University Dongtan Sacred Heart Hospital, Hwaseong, Republic of Korea | First Author | Study concept and design; analysis and interpretation of data; and drafting and critically revising the manuscript for intellectual content |
| Min Soo Byun, MD, PhD | Medical Research Center Seoul National University, Seoul, Republic of Korea | Author | Acquisition, analysis and interpretation of data; and critically revising the manuscript for intellectual content |
| Dahyun Yi, PhD | Medical Research Center Seoul National University, Seoul, Republic of Korea | Author | Acquisition, analysis and interpretation of data; and critically revising the manuscript for intellectual content |
| Jun Ho Lee, MD | National Center for Mental Health, Seoul, Republic of Korea | Author | Acquisition, analysis and interpretation of data; and critically revising the manuscript for intellectual content |
| Min Jung Kim, MD | Seoul National University Hospital, Seoul, Republic of Korea | Author | Acquisition, analysis and interpretation of data; and critically revising the manuscript for intellectual content |
| Gijung Jung, RN, PhD | Seoul National University Hospital, Seoul, Republic of Korea | Author | Acquisition, analysis and interpretation of data; and critically revising the manuscript for intellectual content |
| Jun-Young Lee, MD, PhD | SMG-SNU Boramae Medical Center, Seoul National University College of Medicine, Seoul, Republic of Korea | Author | Acquisition, analysis and interpretation of data; and critically revising the manuscript for intellectual content |
| Koung Mi Kang | Seoul National University Hospital, Seoul, Republic of Korea | Author | Acquisition, analysis and interpretation of data; and critically revising the manuscript for intellectual content |
| Chul-Ho Sohn, MD, PhD | Seoul National University Hospital, Seoul, Republic of Korea | Author | Acquisition, analysis and interpretation of data; and critically revising the manuscript for intellectual content |
| Yun-Sang Lee, PhD | Seoul National University College of Medicine, Seoul, Republic of Korea | Author | Acquisition, analysis and interpretation of data; and critically revising the manuscript for intellectual content |
| Yu Kyeong Kim, MD, PhD | SMG-SNU Boramae Medical Center, Seoul, Republic of Korea | Author | Acquisition, analysis and interpretation of data; and critically revising the manuscript for intellectual content |
| Dong Young Lee, MD, PhD | Seoul National University Hospital, Seoul National University College of Medicine, Seoul, Republic of Korea | Corresponding Author | Study concept and design; acquisition, analysis and interpretation of data; and drafting and critically revising the manuscript for intellectual content |

**Coinvestigators**

Coinvestigators are listed at links.lww.com/WNL/B134.

| Name | Location | Role | Contribution |
| --- | --- | --- | --- |
| Dong Young Lee, MD, PhD | Seoul National University College of Medicine | Principal Investigator | Designed and conceptualized the cohort study; Led and supervised the cohort study; coordinated communication among study cores and study sites; acquired funding |
| Min Soo Byun, MD, PhD | Medical Research Center Seoul National University | Core PI Clinical & Executive | Supervised and coordinated the Clinical and Executive core of the cohort study |
| Dahyun Yi, PhD | Medical Research Center Seoul National University | Core PI Neuropsychology | Supervised and coordinated the Neuropsychological Core of the cohort study |
| Yu Kyeong Kim, MD, PhD | SMG-SNU Boramae Medical Center | Core PI PET | Supervised and coordinated the PET Core of the cohort study |
| Chul-Ho Sohn, MD, PhD | Seoul National University College of Medicine | Core PI MRI | Supervised and coordinated the MRI Core of the study |
| Inhee Mook-Jung, PhD | Seoul National University College of Medicine | Core PI Biomarker | Supervised and coordinated the Biomarker Core of the study |
| Murim Choi, PhD | Seoul National University | Core PI Genetics | Supervised and coordinated the Genetic Core of the study |
| Yu Jin Lee, MD, PhD | Seoul National University College of Medicine | Core PI Sleep | Supervised and coordinated the Sleep Core of the study |
| Seokyung Hahn, PhD | Seoul National University College of Medicine | Core PI Biostatistics | Supervised and coordinated the Biostatistics Core of the study |
| Hyun Jung Kim, MD | Changsan Convalescent Hospital | co-investigator | Performed clinical assessment of participants and quality control of the clinical data |
| Mun Young Chang, MD | Chung-Ang University College of Medicine | co-investigator | Coordinated an add-on study of the main cohort study |
| Seung Hoon Lee, MD | Daerim St. Mary's Hospital | co-investigator | Performed clinical assessment of participants and quality control of the clinical data |
| Na Young Han, MD | Dongrae Medical Center | co-investigator | Performed clinical assessment of participants and quality control of the clinical data |
| Jisoo Pae, MD, PhD | Genome & Company | co-investigator | Coordinated an add-on study of the main cohort study |
| Hansoo Park, MD, PhD | Genome & Company | co-investigator | Coordinated an add-on study of the main cohort study |
| Jee Wook Kim, MD, PhD | Hallym University Dongtan Sacred Heart Hospital | co-investigator | Coordinated a study site and performed participants recruitment and quality control of the clinical data |
| Young Min Choe, MD | Hallym University Dongtan Sacred Heart Hospital | co-investigator | Performed recruitment and clinical assessment of participants and monitoring of the clinical data |
| Jong-Min Lee, PhD | Hanyang University | co-investigator | Coordinated an add-on study of the main cohort study |
| Dong Woo Lee, MD, PhD | Inje University Snaggye Paik Hospital | co-investigator | Coordinated a study site and recruited participants of the cohort study |
| Bo Kyung Sohn, MD | Inje University Snaggye Paik Hospital | co-investigator | Coordinated a study site and recruited participants of the cohort study, performed clinical data analysis |
| Seok Woo Moon, MD, PhD | Konkuk University Chungju Hospital | co-investigator | Coordinated a study site and performed clinical data analysis |
| Seung-Ho Ryu, MD, PhD | Konkuk University Medical Center | co-investigator | Coordinated a study site and recruited participants |
| Man Ho Choi, PhD | Korea Institute of Science and Technology | co-investigator | Supervised and coordinated the MRI Core of the study |
| Hyewon Baek, MD | Kyunggi Provincial Hospital for the Elderly | co-investigator | Performed clinical assessment of participants and quality control of the clinical data |
| Yoon-Keun Kim, MD, PhD | MD Healthcare Inc. | co-investigator | Coordinated an add-on study of the main cohort study |
| Kang Ko, MD | National Center for Mental Health | co-investigator | Performed clinical assessment of participants and quality control of the clinical data |
| Jong-Won Kim, MD, PhD | Samsung Medical Center | co-investigator | Supervised and performed genetic analysis |
| Shin Gyeom Kim, MD, PhD | Soonchunhyang University Hospital Bucheon | co-investigator | Coordinated a study site and performed clinical data analysis |
| Sun-Ho Han, PhD | Seoul National University | co-investigator | Coordinated blood sample repository, performed blood-biomarker-related analysis |
| Joo-Youn Cho, PhD | Seoul National University | co-investigator | Coordinated and performed blood-biomarker-related analysis |
| Jae Sung Lee, PhD | Seoul National University | co-investigator | Coordinated and performed PET image data-related analysis |
| Yun-Sang Lee, PhD | Seoul National University | co-investigator | Coordinated the acquisition of the PET data and related logistics |
| Jong Inn Woo, MD, PhD | Seoul National University | co-investigator | Supervised and advised the cohort study |
| Sang Eun Kim, MD, PhD | Seoul National University Bundang Hospital | co-investigator | Coordinated the production of PET radiotracer |
| Byung Chul Lee, PhD | Seoul National University Bundang Hospital | co-investigator | Coordinated the production of PET radiotracer |
| Gi Jeong Cheon, MD, PhD | Seoul National University Hospital | co-investigator | Coordinated the acquisition of the PET data |
| Koung Mi Kang, MD | Seoul National University Hospital | co-investigator | Participated in the acquisition and clinical interpretation of the MRI/MRA data |
| Jee-Eun Park, MD, PhD | Seoul National University Hospital | co-investigator | Performed clinical and sleep-related data analysis |
| Hyeong Gon Yu, MD, PhD | Seoul National University Hospital | co-investigator | Coordinated an add-on study of the main cohort study |
| Jun-Young Lee, MD, PhD | SMG-SNU Boramae Medical Center | co-investigator | Coordinated a study site and performed participants recruitment |
| Hyo Jung Choi, MD |  | co-investigator | Performed clinical assessment of participants and quality control of the clinical data |
| Kwangsoo Kim, Ph.D | Seoul National University Hospital | co-investigator | Supervised and performed biostatistics data analysis |
| Jun Ho Lee, MD | Seoul National University Hospital | co-investigator | Coordinated participant recruitment and follow-up, performed clinical assessment of participants, quality control of the clinical data analysis |
| Sung Wook Park, MD, PhD | Seoul National University Hospital | research fellow | Performed an add-on study and data analysis |
| So Yeon Jeon, MD | Seoul National University Hospital | research fellow | Coordinated participant recruitment and follow-up, performed clinical assessment of participants, quality control of the clinical data |
| Woo Jin Kim, MD, PhD | Seoul National University Hospital | research fellow | Performed clinical assessment of participants and quality control of the clinical data |
| Hak Young Kim | Seoul National University Hospital | psychologist | Performed neuropsychological assessment of participants, quality control and preprocessing of the data |
| Haejung Joung | Seoul National University Hospital | psychologist | Performed neuropsychological assessment of participants. quality control and preprocessing of the data |
| Younghwa Lee | Seoul National University Hospital | Psychologist | Performed neuropsychological assessment of participants, quality control and preprocessing of the data |
| Donghwi Hwang | Seoul National University | image analyst | Performed PET data analysis |
| Seung Kwan Kang | Seoul National University | image analyst | Performed PET data analysis |
| Seong A Shin | Seoul National University | image analyst | Performed PET data pre-processing |
| Jeong Yeon Hwang, MD | Seoul National University | data analyst | Performed sleep-related data analysis |
| Jong-Chan Park | Seoul National University | data analyst | Performed blood-biomarker related analysis |
| Jong-Ho Park | Samsung Medical Center | genetic data analyst | Performed genetic data analysis |
| Jieun Seo | Seoul National University | genetic data analyst | Performed genetic data analysis |
| Gi Jung Jung | Seoul National University Hospital | research coordinator | Coordinated participants recruitment, follow-up and assessment among sites, performed clinical assessment of participants and data monitoring |
| Min Jeong Kim | Seoul National University Hospital | research coordinator | Coordinated participants recruitment, Performed clinical assessment of participants |
| Han Na Lee | Seoul National University Hospital | research coordinator | Coordinated participants recruitment, follow-up and assessment among sites, performed clinical assessment of participants and data monitoring |
| Yun Jung Hwang | Seoul National University Hospital | researcher | Performed the clinical data analysis |
| Joon Hyung Jung, MD | Seoul National University Hospital | researcher | Performed the clinical data analysis |
| Kiyoung Sung, MD | Seoul National University Hospital | researcher | Performed the clinical data analysis |
| Eun Hye Kim | Seoul National University | research assistant | Coordinated and performed the collection and pre-processing of blood samples |
| Han Byul Choi | National Research Center for Dementia | administrative staff | Coordinated participant recruitment and provided administrative support |
